# A genetically encoded system for oxygen generation in living cells

**DOI:** 10.1073/pnas.2207955119

**Published:** 2022-10-10

**Authors:** Andrew L. Markhard, Jason G. McCoy, Tsz-Leung To, Vamsi K. Mootha

**Affiliations:** ^a^Howard Hughes Medical Institute and Department of Molecular Biology, Massachusetts General Hospital, Boston, MA 02114;; ^b^Broad Institute of MIT and Harvard, Cambridge, MA 02142;; ^c^Department of Systems Biology, Harvard Medical School, Boston, MA 02115

**Keywords:** oxygen, chlorite, Cld, SLC5A5, SNORCL

## Abstract

Oxygen plays a key role in supporting life on our planet. It is particularly important in higher eukaryotes where it boosts bioenergetics as a thermodynamically favorable terminal electron acceptor and has important roles in cell signaling and development. Many human diseases stem from either insufficient or excessive oxygen. Despite its fundamental importance, we lack methods with which to manipulate the supply of oxygen with high spatiotemporal resolution in cells and in organisms. Here, we introduce a genetic system, SupplemeNtal Oxygen Released from ChLorite (SNORCL), for on-demand local generation of molecular oxygen in living cells, by harnessing prokaryotic chlorite O_2_-lyase (Cld) enzymes that convert chlorite (ClO_2_^−^) into molecular oxygen (O_2_) and chloride (Cl^−^). We show that active Cld enzymes can be targeted to either the cytosol or mitochondria of human cells, and that coexpressing a chlorite transporter results in molecular oxygen production inside cells in response to externally added chlorite. This first-generation system allows fine temporal and spatial control of oxygen production, with immediate research applications. In the future, we anticipate that technologies based on SNORCL will have additional widespread applications in research, biotechnology, and medicine.

## Significance

Oxygen is one of the most important molecules in living systems, playing a key role in energy metabolism, cellular signaling, and disease. At present, we have few if any ways to manipulate molecular oxygen in living cells with high spatiotemporal control. Here, we introduce a genetic strategy for generating oxygen inside human cells, by simultaneously expressing a transporter and a bacterial enzyme. Together, these proteins promote the uptake of chlorite into the cell and enzymatically produce oxygen. We call this genetic technology SupplemeNtal Oxygen Released from ChLorite (SNORCL). This technology will allow investigation of the effects of short, local pulses of oxygen in cells and tissues. Optimized versions of the technology could have direct medical applications.

Oxygen is vital for life and is one of the most widely used substrates in all of biochemistry (1). One of the most important events for life on our planet was the great oxygenation event (GOE), some 2.1 to 2.4 billion years ago (2), which changed our environment and spawned aerobic life. Oxygen provides a thermodynamically favorable terminal electron acceptor that helps to power metabolism and has been proposed as a prerequisite for the emergence of complex forms of animal life (3). Since oxygen is a di-radical and can be toxic, numerous mechanisms evolved to allow organisms to safely wield its thermodynamic potential (4). In addition, oxygen plays a key role in signaling (5, 6) and contributes to cell differentiation and development (7). Humans have an absolute requirement for oxygen, being only able to survive minutes in complete anoxia. At the other extreme, hyperoxia can also be toxic, leading to seizures, pulmonary toxicity, and retinopathy (8).

Blood oxygen levels are routinely monitored in clinical medicine, and when required, we have facile means of delivering supplemental oxygen through nasal cannula, face masks, mechanical ventilation, and even extracorporeal membrane oxygenation. In contrast, we only have few ways of providing supplemental oxygen within cells. In the research setting, cells and organisms of course can be grown in chambers in which the ambient oxygen is regulated with gas mixtures (8). However, the poor solubility of oxygen in biofluids, its continuous exchange with the atmosphere, and its active consumption by mitochondrial respiration, make it challenging to manipulate intracellular oxygen levels with high spatiotemporal precision. Ideally, we would have an easy-to-use, genetically encoded system capable of delivering on-demand, localized production of molecular oxygen in living cells.

Here we sought to develop such a tool by harnessing naturally occurring enzymes that generate molecular oxygen. While genetic tools exist for generating reactive oxygen species such as singlet oxygen in living cells (9), none have been described that generate molecular oxygen in its more familiar and stable triplet state. Enzymatic formation of the O-O bond is extremely rare. The most well appreciated and studied example is the water-splitting oxygen evolving complex (OEC) of photosystem II, which is central to oxygenic photosynthesis. However, the OEC contains numerous cofactors including chlorophyll, quinones, and a unique manganese cluster (10). Oxygen can also be produced from methane-oxidizing bacteria (11), though the mechanism is not well studied. Another enzyme, called chlorite O_2_-lyase or chlorite dismutase (Cld), converts chlorite (ClO_2_^−^) to oxygen (O_2_) and chloride (Cl^−^) in numerous bacterial and archaeal species [reviewed in ref. (12)].

We chose to focus on the Cld family of oxidoreductases as a chassis for a simple-to-use oxygen generator given that its substrate is bioorthogonal to eukaryotic metabolism. We show that when expressed in human cells, Cld enzymes exhibit high activity, and that we can coexpress plasma membrane transporters that promote uptake of sodium chlorite for its subsequent intracellular conversion to oxygen. In this way we are able to successfully deploy a genetic system for SupplemeNtal Oxygen Released from ChLorite (SNORCL).

## Results

**Fig. 1.**
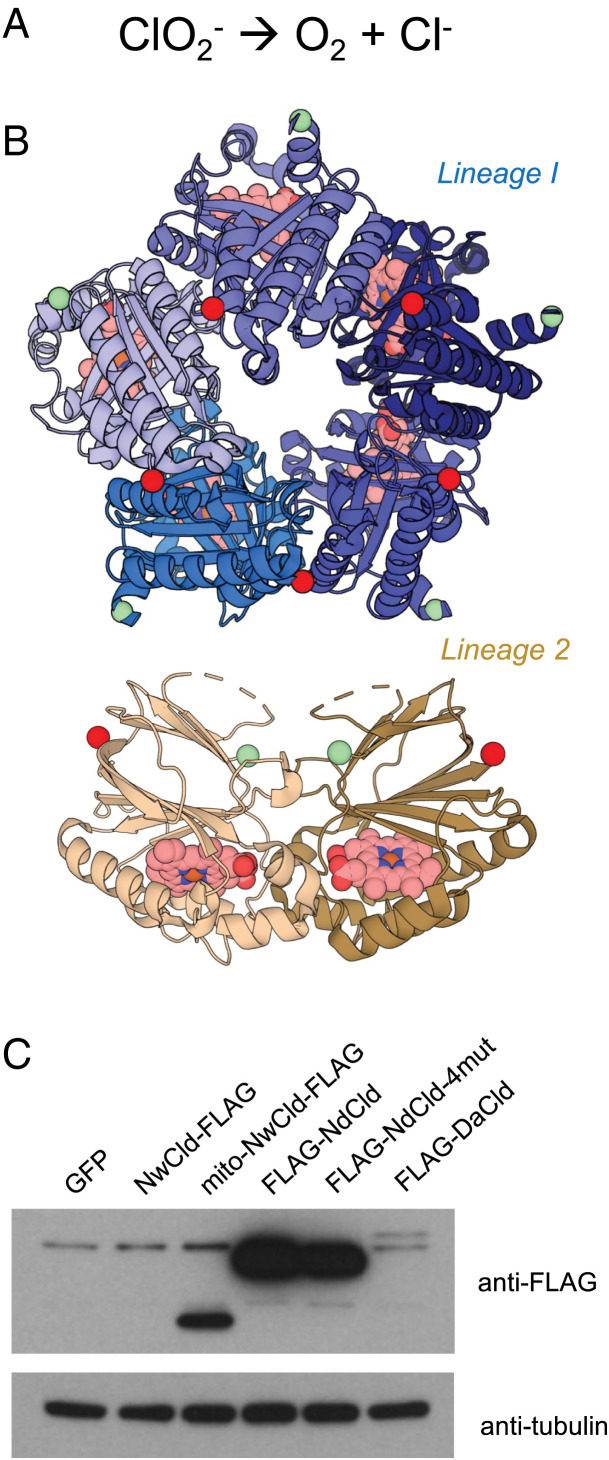
Screening Cld variants for expression in human cells. (*A*) Reaction catalyzed by the Cld enzymes. (*B*) Structures of representative Cld enzymes from *Nd* (Lineage I) and *Nw* (Lineage II) (PDB accession # 3NN2 and 3QPI; *N*- and C-termini are represented with green and red spheres, respectively; the heme *b* cofactors are colored pink). (*C*) Lysates from HeLa cells transduced with lentivirus for indicated constructs were subjected to SDS-PAGE and immunoblotted to confirm expression of FLAG-tagged Cld variants or loading control.

Cld oxidoreductases (EC 1.13.11.49) are distributed in bacteria and archaea and were originally discovered in 1996 in perchlorate respiring organisms (13). These enzymes catalyze the conversion of ClO_2_^−^ to oxygen and chloride (Fig. 1*A*). Cld enzymes can be homo-pentameric (Lineage I, found in *Dechloromonas aromatica* and *Nitrospira defluvii*) or homo-dimeric (Lineage II, found in *Nitrobacter winogradskyi*) (Fig. 1*B*). All characterized Cld enzymes possess an iron-containing heme *b* cofactor with histidine as the axial ligand, as well as a highly conserved arginine critical for catalysis (reviewed in (12)). Cld enzymes tend to be fast, do not generate reactive oxygen species, and can exhibit high turnovers before inactivating (14). Although purified Cld enzymes have been proposed for in vitro detoxification or for studying rapid in vitro kinetics of oxygen-dependent enzymes (15), to our knowledge, no prior studies have proposed expressing them within eukaryotic cells for oxygen production.

We began by testing the expression of several naturally occurring Cld variants as well as ones we engineered for greater thermostability or subcellular localization. To facilitate expression and purification from human HeLa cells, Cld genes were engineered through codon optimization, deletion of predicted periplasmic targeting sequences, and incorporation of epitope tags at the termini least likely to impact enzyme activity as suggested by published pentameric and dimeric CLD structures. We tested enzymes from both lineage 1 (FLAG-*Nd*Cld, FLAG-*Da*Cld) and lineage 2 (*Nw*Cld-FLAG), including one targeted to mitochondria (mito-*Nw*Cld-FLAG). We also used computational methods (16) to design four point mutations predicted to improve *Nd*Cld thermostability (FLAG-*Nd*Cld^4xMUT^). In these preliminary screens we saw the greatest expression from N-terminally FLAG-tagged *Nd*Cld (Fig. 1*C*), which became the focus of our study. Cells expressing FLAG-*Nd*Cld appeared healthy, comparable to cells expressing green fluorescent protein (GFP), without any obvious impact on cell morphology or growth.

**Fig. 2.**
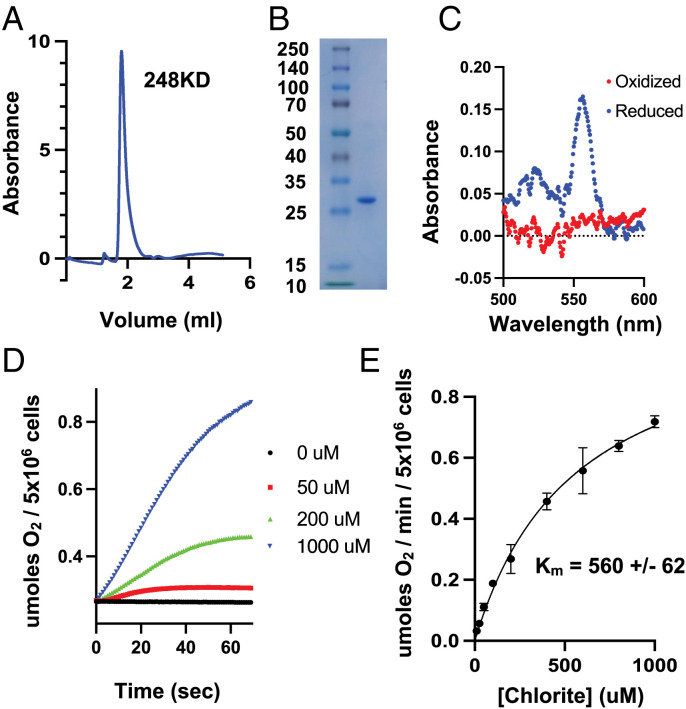
Cld expressed in human cells assembles properly with high activity. (*A*) Size exclusion chromatography profile of purified *Nd*Cld. (*B*) SDS-PAGE analysis of purified *Nd*Cld visualized with coomassie. (*C*) Absorption spectra of purified *Nd*Cld in the presence of 500 μM ferricyanide with (reduced) or without (oxidized) 2.5 mM dithionite. (*D*) Representative time traces of molecular oxygen formation with different chlorite concentrations. (*E*) Steady-state enzyme kinetics of *Nd*Cld catalyzed oxygen production. Shown is the mean ± SEM of *n* = 4 measurements.

We then sought to determine whether FLAG-*Nd*Cld expressed in HeLa cells grown in standard cell culture conditions was properly assembled with its heme *b* cofactor. We cultured cells expressing FLAG-*Nd*Cld and performed affinity purification under nondenaturing conditions. The purified enzyme is monodispersed, as shown by gel filtration chromatography, running at an apparent molecular weight of 248 kDa (Fig. 2*A*). Sodium dodecyl sulfate-polyacrylamide gel electrophoresis (SDS-PAGE) analysis results in a clean, Coomassie-stained band at the expected molecular weight of 29 kDa (Fig. 2*B*). Given that *Nd*Cld, as well as other Lineage I Clds, is known to form pentamers (17), we speculate that the enzyme is running as a dimer of pentamers. The oxidized and reduced spectra, obtained by addition of ferricyanide or dithionite, respectively, confirm that the enzyme expressed in human cells incorporates a heme *b* cofactor (Fig. 2*C*). We quantified the heme concentration from the absorbance at 557 nm of the reduced *Nd*Cld sample. By assuming a heme extinction coefficient of 34.7 mM^−1^cm^−1^ (18), we estimate 98% incorporation of heme in the purified FLAG-*Nd*Cld.

Next, we characterized the activity of this protein in human cell extracts. We permeabilized HeLa cells with digitonin and then performed a dose-response experiment with addition of sodium chlorite. Doses spanning 10 μM to 1 mM were used, as previous studies have shown that higher chlorite concentrations lead to inactivation of the enzyme (19). We monitored oxygen levels using an optical oxygen meter in a well-stirred, air-saturated cuvette. We observe a very rapid oxygen evolution in response to added sodium chlorite, as shown in representative traces in Fig. 2*D*, consistent with what has been reported for bacterially expressed and purified enzyme. For example, with 5 million cells in a well-stirred 1 mL cuvette, a pulse of 1 mM sodium chlorite leads to generation of 359 ± 10 nmol O_2_ over the first 30 seconds. For the bacterially expressed *Nd*Cld, a broad range of *K_m_* values have been reported, ranging from 58 to 69 μM for the purified enzyme (17), [reviewed in ref. (12)], to as high as 15.8 mM in the original characterization of *Nd*Cld in *Escherichia coli* extracts (20). We estimate that the *K_m_* for chlorite is 560 μM (Fig. 2*E*). The apparent rate of oxygen production by *Nd*Cld in our system is quite reasonable, given that HEPES and chloride can inhibit Cld activity (21, 22), energized intact mitochondria in our extracts continue to consume oxygen, and rapidly generated oxygen may equilibrate with the atmosphere. These studies demonstrate that *Nd*Cld enzymes can be safely expressed in human cells grown in standard cell culture conditions. They oligomerize, fully incorporate the heme *b* cofactor, and in permeabilized extracts, function properly with rapid production of oxygen from chlorite.

**Fig. 3.**
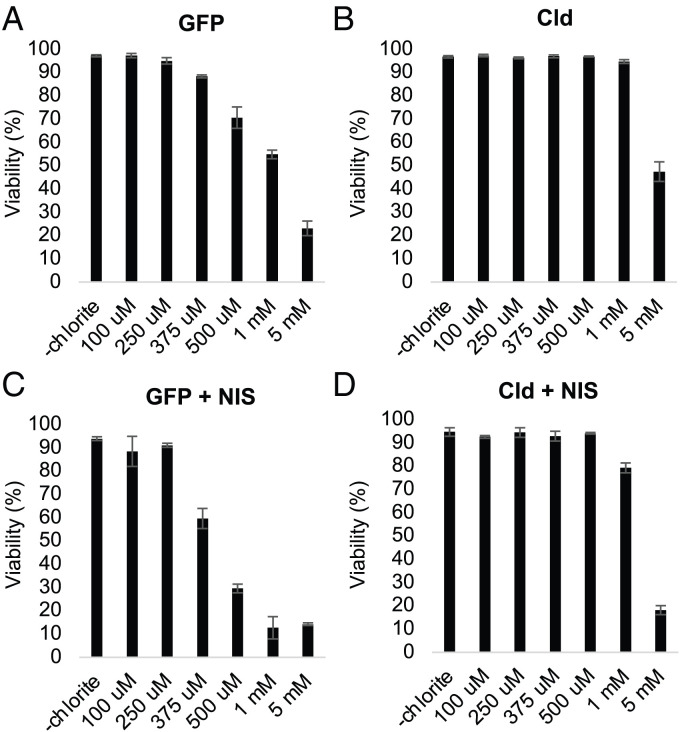
Three-day toxicity of sodium chlorite in human HeLa cells. HeLa cells expressing (*A*) GFP, (*B*) *Nd*Cld, (*C*) GFP + NIS, or (*D*) *Nd*Cld + NIS were grown for 3 d with the indicated concentration of freshly prepared sodium chlorite. Cell counts and viability were assessed after 3 d of growth using a Vi-Cell BLU Cell Viability Analyzer. Shown is the mean ± SD of triplicate measurements.

For *Nd*Cld to be useful as an oxygen-producing enzyme in intact cells, sufficient sodium chlorite would have to be imported across the plasma membrane. As chlorite is negatively charged and polar, it is not expected a priori to passively diffuse across a lipid bilayer. Nonetheless, previous studies have shown that at very high doses, chlorite compromises fitness and growth of cells due to its oxidant properties (23). For example, a 4 mM dose can achieve 50% growth inhibition in yeast (24). In HeLa cells, we observed a 50% decrease in viability when cells were treated with ∼2 mM of sodium chlorite for 3 d (Fig. 3*A*). However, the toxicity was alleviated by the expression of *Nd*Cld (Fig. 3*B*). These data suggest that at a high dose chlorite can enter HeLa cells over a 3-d period, and that it is toxic in a way that can be alleviated by expression of *Nd*Cld.

Using this 3-d toxicity assay, we screened for transporters that might promote further uptake of chlorite into human cells. To our knowledge, no study has ever investigated chlorite transport, although transport activity for other polyatomic anions such as nitrate, nitrite, and chlorate have been reported. In pilot studies, we expressed nitrate transporters from plants, fungi, and humans, and observed no obvious boost in chlorite toxicity. We then turned to the human sodium iodide symporter (NIS), encoded by *SLC5A5* (25). The human NIS is expressed as a homodimer on the basolateral membrane of thyroid follicular cells, where it concentrates iodide with symport of two Na^+^ ions. Electrophysiological studies of NIS in *Xenopus* oocytes show that it has broad transport activity for many polyatomic anions, including chlorate (ClO_3_^−^) with a *K_m_* of 277 μM (25). When we expressed human NIS in HeLa cells, we observed a five-fold increase in the 3-d toxicity of added sodium chlorite (Fig. 3*C*), that could be attenuated by *Nd*Cld expression (Fig. 3*D*). The most parsimonious explanation of our results is that NIS promotes sodium chlorite uptake into the HeLa cells, and *Nd*Cld detoxifies the chlorite by catalyzing its conversion into molecular oxygen and chloride.

**Fig. 4.**
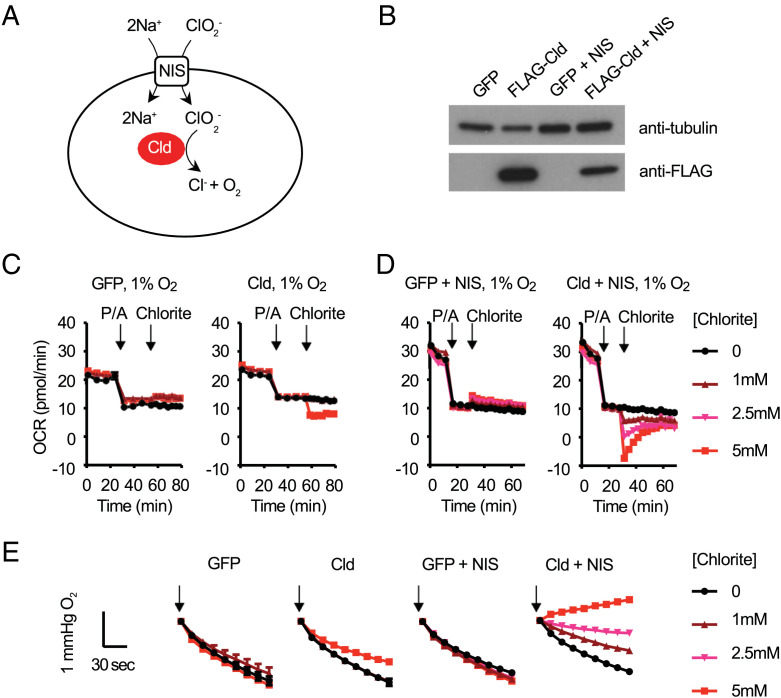
On-demand generation of oxygen using SNORCLs in intact human cells. The Agilent Seahorse XFe96 system was used to measure oxygen levels and oxygen consumption rates (OCR) in live, intact HeLa cells. (*A*) Overview of the SNORCL system and reaction catalyzed by Cld and transport facilitated by NIS. (*B*) Western blot of FLAG-*Nd*Cld in HeLa cells. (*C*) Seahorse intact cell oxygen consumption rate measurements at 1% ambient oxygen with sequential additions of piericidin+antimycin (1 μM each) and sodium chlorite (0, 1 mM, or 5 mM) in HeLa cells expressing GFP or FLAG-*Nd*Cld. (*D*) Seahorse intact cell oxygen consumption rate measurements at 1% ambient oxygen with sequential additions of piericidin+antimycin (1 μM each) and sodium chlorite (0, 1 mM, 2.5 mM, or 5 mM) in HeLa cells expressing GFP + NIS or FLAG-*Nd*Cld + NIS. (*E*) Traces of the oxygen levels within two minutes upon sodium chlorite addition (black arrow) in the Seahorse experiments shown in Fig. 4 *C* and *D*. Mean ± SEM of *n* = 4 to 6 biological replicates are shown in Fig. 4 *C*–*E*.

We sought to determine whether we could detect SNORCL-dependent oxygen evolution in intact cells (Fig. 4*A*). We grew HeLa cells expressing either FLAG-*Nd*Cld or GFP, with or without the coexpression of NIS (Fig. 4*B*) and monitored the chlorite-dependent oxygen production by measuring the oxygen consumption rate (OCR) with the Seahorse XFe96 Analyzer. We anticipated challenges in being able to detect oxygen evolution by SNORCLs given that any oxygen it generates could rapidly equilibrate with the atmosphere, and second, mitochondria could actively consume it. After initial experiments at atmospheric 21% oxygen (*SI Appendix*, Fig. S1 *A* and *B*), where we did observe modest but reproducible oxygen generation in a Cld-dependent manner, we performed subsequent experiments in a 1% ambient oxygen environment (to prevent back diffusion) while treating cells with piericidin and antimycin (to block mitochondrial respiration).

Under these conditions, oxygen generation, as evidenced by a decline in apparent oxygen consumption rate (OCR), was immediately obvious and striking in cells coexpressing both *Nd*Cld and NIS, with a clear dose-response beginning with added chlorite (Fig. 4 *C* and *D*). In these experiments, maximal rates of oxygen evolution occurred during the first few minutes, which tapered off but continued for more than 30 min. Cells remained viable throughout the course of these experiments, as measured at 1 h after the addition of even the highest dose of chlorite (*SI Appendix*, Fig. S1*C*). Partial pressure of oxygen reported by the Seahorse instrument (Fig. 4*E*) clearly shows a chlorite dose-dependent oxygen evolution in these intact cells in a way that is boosted by coexpressing NIS (Fig. 4*E*).

To further confirm that oxygen generation was taking place inside the cell, we performed an independent set of experiments in which we measured oxygen with both permeabilized and intact cells (*SI Appendix*, Fig. S2). In this set of experiments, we again expressed FLAG-*Nd*Cld, and this time coexpressed either NIS or mCherry, the latter serving as a viral transduction and antibiotic selection control. In both cell lines, we saw robust protein expression of FLAG-*Nd*Cld (*SI Appendix*, Fig. S2*A*). When the plasma membrane is permeabilized, both cell lines exhibit comparable dose–responses to injected sodium chlorite (*SI Appendix*, Fig. S2*B*). In intact cells, although we were able to generate oxygen pulses in both cell lines at a high dose of 5 mM chlorite, we observed oxygen evolution even with 500 μM or 1 mM of chlorite in NIS but not mCherry expressing cells (*SI Appendix,* Fig. S2 *C* and *D*). These studies further confirm that coexpressing the NIS facilitates the transport of the chlorite into cells.

**Fig. 5.**
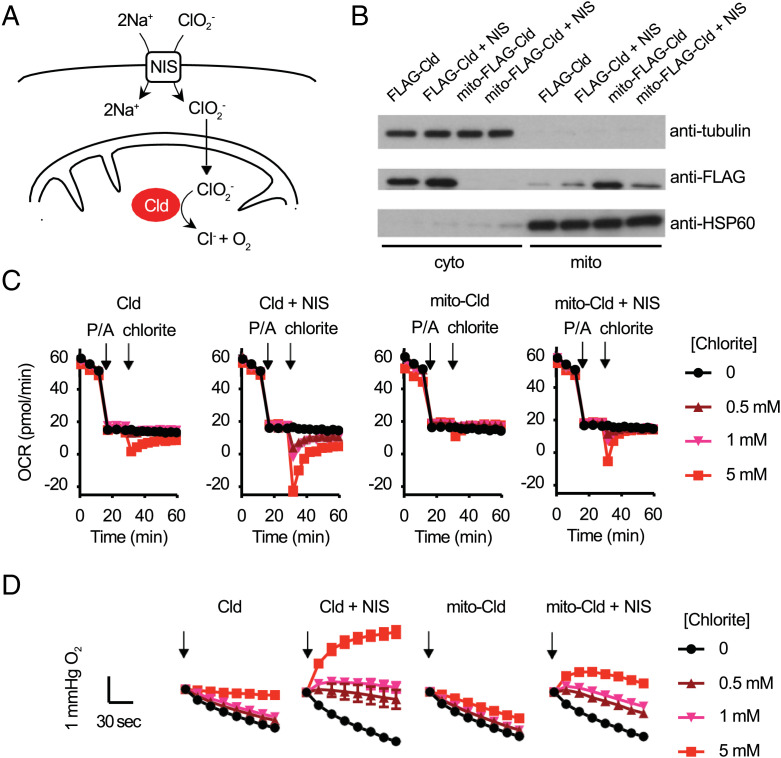
Subcellular targeting of SNORCLs to generate oxygen in the cytosol or mitochondria. (*A*) Overview of mitochondrial targeted SNORCL. (*B*) Immunoblots of mitochondrial and cytosolic fractions expressing FLAG-*Nd*Cld or mito-FLAG-*Nd*Cld. (*C*) Seahorse intact cell oxygen consumption rate measurements at 1% ambient oxygen with sequential additions of piericidin+antimycin (1 μM each) and sodium chlorite (0, 0.5 mM, 1 mM, or 5 mM) in HeLa cells expressing FLAG-*Nd*Cld or mito-FLAG-*Nd*Cld, with or without NIS. (*D*) Raw traces of the oxygen levels within 2 min upon sodium chlorite addition (black arrow) in the Seahorse experiments shown in Fig. 5*C*. Mean ± SEM of *n* = 4 to 6 biological replicates are shown in Fig. 5 *C* and *D*.

Finally, we sought to determine whether we could genetically target the SNORCL system to different subcellular compartments (Fig. 5*A*). We introduced an N-terminal mitochondrial targeting sequence to FLAG-*Nd*Cld (mito-FLAG-*NdCld*) and compared it to FLAG-*Nd*Cld. These constructs successfully targeted the enzyme to mitochondria and the cytosol, respectively, based on immunoblot analysis of respective cell fractions (Fig. 5*B*). To determine whether the mitochondrial targeted *Nd*Cld can function, we performed Seahorse analysis in intact cells, and found that the mito-FLAG-*NdCld* is also capable of generating oxygen in response to added chlorite (Fig. 5*C*). Examination of the oxygen partial pressures from the Seahorse instrument (Fig. 5*D*) confirms net generation of oxygen by mito-FLAG-*Nd*Cld when NIS is coexpressed. These experiments support that chlorite entering into the cell is able to be taken up by mitochondria, presumably via mitochondrial anion transporters. Collectively these studies provide proof that SNORCL can be targeted to mitochondria to permit on-demand oxygen generation with spatiotemporal resolution.

## Discussion

Here we have introduced the SNORCL technology for the facile generation of molecular oxygen within living cells. Although oxygen is critical for life, at present, we have few or no means of manipulating intracellular oxygenation inside cells or organisms with precise genetic control. Oxygen tensions can vary tremendously across mammalian organs and within tissues (8), and a theoretical 1,000-fold gradient can exist between capillaries and mitochondria (26). The current state of the art for manipulating molecular oxygen entails placing cell culture plates or organisms in chambers in which the ambient oxygen tension can be controlled through the mixture of gases. Although a variety of genetic tools exist for generating reactive oxygen species such as singlet oxygen (9), to our knowledge SNORCL is the first genetic tool for generating molecular oxygen inside cells.

SNORCL adds to a growing set of genetic tools with which to manipulate metabolism. We previously introduced a naturally occurring enzyme, *Lb*NOX, for organelle-specific manipulation of the NADH/NAD^+^ ratio in living human cells (27). We subsequently used protein design to engineer a quintuple mutant of *Lb*NOX to create triphosphopyridine nucleotide oxidase (TPNOX), a synthetic, highly specific water-forming NADPH oxidase for compartment specific manipulation of the NADPH/NADP^+^ ratio (28). These tools have even been taken into in vivo mouse studies, laying the technical foundation for the emerging field of causal metabolism (29, 30). As oxygen is one of the most important and widely used molecules that powers metabolism and serves as a trigger for many cellular signaling programs, having a genetic means for precise oxygen manipulation promises to be valuable.

Our first generation SNORCL is capable of producing pulses of oxygen in intact human cells that last on the order of many minutes. We have measured SNORCL-mediated oxygen evolution in intact and permeabilized cells using the Seahorse system. However, care must be taken in quantitative interpretion of these rates of oxygen evolution. The Seahorse apparatus utilizes a phosphorescent oxygen sensor at the tip of a plunger that is intermittently submerged in the sample well to create a small-volume microchamber inside which OCR is estimated. This plunging also serves to mix the media following injection of chemicals such as chlorite in our case. Given the very rapid initial kinetics of Cld enzymes and the ability of the evolved oxygen to equilibrate with the media before the microchamber is formed, initial rates of oxygen evolution may not be accurately estimated with Seahorse. With these caveats, our back of the envelope estimates indicate that we are able to evolve oxygen in permeabilized cells at rates comparable to resting oxygen consumption in HeLa cells, while in intact cells, by utilizing NIS to catalyze chlorite transport, we observe oxygen evolution rates ∼1/10th this value.

We acknowledge two limitations of our first generation SNORCL technology. First, the Cld enzyme is known to self-inactivate, although oxygen evolution can be robust on the timescale of minutes to an hour depending on the chlorite concentration. This inactivation has been shown to result from bleaching of the heme that may be caused by the escape of a reaction intermediate from the enzyme active site (19). Second, chlorite is an oxidant, and although it can often be found in drinking water, it can be toxic at high doses (31, 32). Here we leveraged the use of the NIS transporter to promote chlorite uptake and could demonstrate SNORCL-mediated generation of intracellular oxygen using doses of 500 μM to 1 mM (Fig. 5 and *SI Appendix*, Fig. S2), which are well tolerated by Cld expressing cells (Fig. 3). Initial application of SNORCL technology must be cognizant of the magnitude/duration of oxygen generation and potential off-target effects of chlorite. Appropriate use of controls (e.g., testing +/−Cld, +/−NIS) can help to ensure that any observed effects are directly attributable to the generated oxygen. With additional protein engineering of the enzyme and the transporter, both of these limitations can be overcome to enable more chronic in vivo studies.

We anticipate technologies based on SNORCL could enjoy many applications. In the research arena, SNORCL represents a new genetic tool with which to evaluate the causal role of localized oxygen in signaling, metabolism, and physiology with unprecedented spatiotemporal resolution. In the medical arena, we could envision delivering SNORCL systems as a gene therapy to target tissues and alleviate hypoxia-mediated diseases, or alternatively, it may be useful in boosting the activity of cellular therapies such as CAR-T, where hypoxia in the tumor microenvironment limits its efficacy (33). It is even conceivable that organisms genetically modified to express SNORCL will promote survival in extraterrestrial, anoxic zones where chlorite may be present (34, 35).

## Materials and Methods

### Sequences Used in This Study.

GFP was obtained from Addgene #19319, pLJM1-eGFP. mCherry was from Addgene #32383, pcDNA3.1-Peredox-mCherry. All other sequences were custom designed and synthesized for use in this study and are provided in *SI Appendix*.

### Generation of Cell Lines Stably Expressing Transgenes.

Cld enzymes and sodium/iodide symporters were stably expressed in HeLa cells using lentiviral transduction. Briefly, gene constructs were custom synthesized in pUC57-Kan (GenScript) with NheI and EcoRI restriction sites at the 5′ and 3′ ends, respectively. Cld cDNA was subcloned into the pLYS1 lentiviral expression vector (Addgene #50057), while SLC5A5 cDNA was subcloned into pLYS5 (Addgene #50054). Construct sequences were verified by Sanger sequencing (Azenta). Lentivirus was generated in 293T cells (ATCC #CRL-3216). 10^6^ cells were seeded per dish in 6 cm culture dishes, in 5 mL media. The next day, the cells were transfected using X-tremeGENE HP transfection reagent (Roche #6366244001) with 1 μg of lentiviral construct, along with 900 ng psPAX2 (Addgene #12260) and 100 ng pCMV-VSV-G (Addgene #8454) lentiviral packaging and envelope plasmids. After 48 h, lentivirus was collected and passed through a 0.45 μm polyethersulfone syringe filter (Whatman #6780-2504). For lentiviral transduction, 2 × 10^5^ HeLa cells (ATCC #CCL-2) were seeded for each transduction. The next day, cells were treated with 8 μg/mL polybrene (Sigma #H9268) and transduced with 400 μL lentivirus. After 48 h, cells were passaged and selected with 2 μg/mL puromycin (Gibco #A1113803) or 100 μg/mL hygromycin B (Sigma #H3274), as appropriate. Once fully selected, cells were maintained in puromycin or hygromycin B for an additional passage prior to use for subsequent experiments. HeLa cells were maintained in Dulbecco’s Modified Eagle Medium (DMEM, Gibco #11995-065) supplemented with 10% fetal bovine serum (FBS, Sigma #2442), 1× GlutaMax (Gibco #35050061), and penicillin/streptomycin (Gibco #15140122). Cells were maintained in a 37 °C, 5% CO_2_ incubator.

### Immunoblot Analysis.

For Western blots from HeLa cell lysates, cells were first washed with ice cold PBS, then lysed with ice cold 1% Triton lysis buffer supplemented with protease/phosphatase inhibitor (Cell Signaling #5872). Mitochondrial and cytosolic fractions were prepared as previously described (27). Lysates were clarified by centrifugation at 21,000 × g for 10 min, at 4 °C. Supernatants were transferred to clean microcentrifuge tubes on ice. Protein content was quantified by Bradford assay (Bio-Rad #5000205). Samples were normalized to 1 μg/μL in lysis buffer with 1× SDS sample buffer (2% SDS, 5% β-mercaptoethanol, 5% glycerol, 47.4 mM Tris HCl, 16.6 μM Bromophenol Blue, pH 6.8). Samples were heated for 5 min at 95 °C on a heat block and cooled at room temperature before loading on SDS-PAGE gels. Samples were run on Tris-Glycine gels at 120 V for ∼2 h, then transferred to polyvinylidene difluoride (PVDF) membranes (Bio-Rad #1704157) using a Trans-Blot Turbo Transfer System (Bio-Rad). Membranes were blocked in 5% milk/Tris-Buffered Saline with Tween-20 (TBST) for 1 h at room temperature. Membranes were probed with anti-GFP (Abcam #ab6556), anti-FLAG (Cell Signaling #2368), or anti-β-tubulin (Cell Signaling #2128) diluted 1:1,000 in 5% milk/TBST, incubated overnight at 4 °C. HRP-conjugated donkey anti-rabbit (Cell Signaling #7074) secondary antibody was used at 1:10,000 dilution in 5% milk/TBST for 1 h at room temperature. Membranes were washed 6 × 5 min with 1× TBST before and after secondary antibody incubation. Membranes were incubated with Western Lightning Plus ECL substrate (PerkinElmer #NEL104001EA) for 3 min. Luminescence was detected using Amersham Hyperfilm ECL film (GE Healthcare #28906838) developed on an X-Omat 2000A Processor (Kodak).

### Purification and Biochemical Characterization of *Nd*Cld Expressed in Human Cells.

HeLa cells were harvested, washed in PBS, and resuspended in 300 mM NaCl, 50 mM Hepes pH 7.4, 2% glycerol, complete EDTA-free protease inhibitor mixture (Roche), PMSF, and Benzonase (Millipore Sigma). Cells were lysed with 10 strokes of a tight Dounce homogenizer followed by 90 sec of sonication on ice. The suspension was centrifuged at 25,000 × *g* for 1 h and the resulting lysate was incubated with anti-FLAG M2 affinity gel (Millipore Sigma) for 90 min. The slurry was loaded into a gravity flow column, washed, and then eluted using 3× FLAG peptide. The collected protein was concentrated via Amicon 10 kDa centrifugal filters (Millipore Sigma), filtered, and then loaded onto a Superdex 200 Increase 5/150 GL gel filtration column (Cytiva) equilibrated with 100 mM NaCl, 20 mM Hepes pH 7.4, and 0.2% glycerol. Sizing of the protein through gel filtration was accomplished by comparison to a gel filtration standard (Bio-Rad) run under identical buffer, flow rate, and temperature conditions.

### Assessment of Heme Content in Purified Protein.

Heme incorporation was measured through the pyridine hemochromagen assay (36). Spectra were collected using a Nanodrop OneC (Thermo Fisher). Equal volumes of purified *Nd*CLD (9.4 μM) and 0.2 M NaOH, 40% (vol/vol) pyridine, and 500 μM potassium ferricyanide were mixed to obtain the oxidized spectra followed by 2.5 mM sodium dithionite to obtain the reduced spectra. The heme concentration was then determined from the absorbance at 557 nm of the reduced *Nd*CLD sample using the heme extinction coefficient 34.7 mM^−1^cm^−1^ (18). The calculated heme concentration, 4.6 μM, corresponded to 98% incorporation of heme in the purified *Nd*CLD.

### Steady-State Enzyme Kinetics of *Nd*Cld in Permeabilized Human Cells.

HeLa cells were pelleted at 800× *g* for 3 min, washed with PBS, pelleted again, and then resuspended in assay buffer (125 mM KCl, 2 mM K2HPO4, 1 mM MgCl2, 20 mM Hepes pH7.2, 5 mM glutamate, 5 mM malate, and 0.01% digitonin) at a concentration of 5 × 10^6^ cells/mL. Oxygen production was measured using a FireSting optical oxygen meter connected to a sensor vial (PyroScience GmbH). One ml of cell solution (5 × 10^6^ cells) was used for each measurement. Measurements were performed under ambient air conditions with constant stirring of the cell solution. The reaction was initiated by adding sodium chlorite (prepared in assay buffer). The initial rates were determined from the resulting oxygen traces using up to 20 seconds of the linear portion of the trace via the ICEKAT web server (37). The means of 4 replicate rates were plotted against the chlorite concentrations to estimate the *K_M_*.

### Three-Day Toxicity of Sodium Chlorite in Human Cells.

HeLa cells were trypsinized, counted, and prepared at 10^5^ cells/mL in normal growth media. One molar sodium chlorite stock solution was prepared fresh at the time of the assay in UltraPure ddH2O, and diluted to 2× working concentrations in cell growth media. Cells were seeded in 24-well plates, with triplicate wells for each condition. Five hundred microliters of each 2× chlorite/media preparation was first added to the plate. Five hundred microliters of cell suspension (5 × 10^4^ cells) was then added to each well. The plate was gently mixed, and cells were grown for 3 d in a 37 °C/5% CO_2_ incubator. After 3 d, cells were washed briefly with 500 μL of PBS, trypsinized with 250 μL TrypLE Express, and resuspended with 750 μL of normal growth media to 1 mL total volume. In wells containing a majority of visibly dead, floating cells, cells were resuspended by vigorously pipetting up and down rather than by trypsinization. Two hundred microliters of each cell suspension was then quantified using a Vi-Cell BLU Cell Viability Analyzer (Beckman Coulter).

### Measurement of Oxygen and Oxygen Consumption Rates in Intact or Permeabilized Cells.

For OCR and O_2_ measurements in HeLa cells using the Agilent Seahorse XFe96 system, cells were seeded at 1.5 × 10^4^ cells in 80 μL/well in 96-well Seahorse cell culture plates, in DMEM (Gibco #11995-065) supplemented with 10% fetal bovine serum (FBS; Gibco #26140-079) and penicillin/streptomycin (Gibco #15140-122). After 16 to 20 h, 175 μL of HEPES buffered Seahorse DMEM supplemented with 10 mM glucose, 1 mM sodium pyruvate, and 2 mM L-glutamine (Agilent) was added, and the plate was transferred to a 37 °C non-CO_2_ incubator for 1 h. The Seahorse cartridge was hydrated according to the manufacturer’s protocol. Piericidin A (Enzo Life Sciences) + Antimycin A (Sigma) and sodium chlorite (Sigma) were prepared in Seahorse Dulbecco's Modified Eagle Medium (DMEM) and added to the wells by injections during the Seahorse run. Three or four baseline respiratory rate measurements were taken, followed by sequential injections of Piericidin A+Antimycin A (three or four measurements) and sodium chlorite (12 measurements). To confirm uniform cell numbers across cell lines and no striking changes in cell numbers over the course of a Seahorse experiment (*SI Appendix*, Fig. S1*C*), after the Seahorse run 2 μg/mL Hoechst 33342 (Invitrogen) was added to each well and incubated for 10 min, and the plate was imaged on a BioTek Cytation 5 Cell Imaging Multi-Mode Reader. The total number of nuclei (a proxy for the cell number) in each well was determined. To perform Seahorse measurement at 1% ambient oxygen, a XFe96 system was set up in a Coy O_2_ Control InVitro Glove Box. Hydrated Seahorse cartridge, Seahorse DMEM, and other reagents were incubated at 1% ambient oxygen in the glove box overnight prior to the Seahorse experiment. During the Seahorse run at 1% ambient oxygen, the “Hypoxia mode” was used according to Agilent’s protocol. Freshly prepared sodium sulfite solution was loaded into the cartridge to provide a “zero” oxygen reference.

For permeabilized Seahorse OCR measurements, HeLa cells were seeded at 1.5 × 10^4^ cells/well in 80 μL/well growth media and grown overnight at 37 °C. Seahorse cartridges were hydrated overnight at 37 °C, according to the manufacturer’s protocol. After 16 to 20 h, cells were washed once with mannitol and sucrose (MAS) buffer (70 mM sucrose, 220 mM mannitol, 5 mM KH_2_PO_4_, 5 mM MgCl_2_, 2 mM HEPES, 1 mM ethylene glycol-bis(beta-aminoethyl ether)-*N*,*N*,*N*',*N*'-tetraacetic acid (EGTA), 0.2% fatty acid-free bovine serum albumin [BSA]). Cells were then permeabilized with MAS buffer supplemented with 2 nM XF Plasma Membrane Permeabilizer (Agilent 102504-100) and 1 μM each of Piericidin A+Antimycin A. Upon assay start, six baseline respiratory rate measurements were taken, followed by injection of chlorite and twelve respiratory rate measurements after chlorite injection. Permeabilized Seahorse experiments were also performed at 1% ambient oxygen, in a Coy O_2_ Control InVitro Glove Box as described above.

## Supplementary Material

Supplementary File

## Data Availability

All study data are included in the article and/or supporting information.
