# Supplementary material for: A genetically encoded system for oxygen generation in living cells
Source: Proc Natl Acad Sci U S A. 2022 Oct 10;119(43):e2207955119. doi: 10.1073/pnas.2207955119 (PMC9618058; doi:10.1073/pnas.2207955119)
Supplement: Supplementary File [file pnas.2207955119.sapp.pdf]

PNAS

Supporting Information for

A genetically encoded system for oxygen generation in living cells

Andrew L. Markhard, Jason G. McCoy, Tsz-Leung To, Vamsi K. Mootha

Correspondence: Vamsi K. Mootha
Email: vamsi@hms.harvard.edu

This PDF file includes:
Figures S1-S2
Supplementary Methods

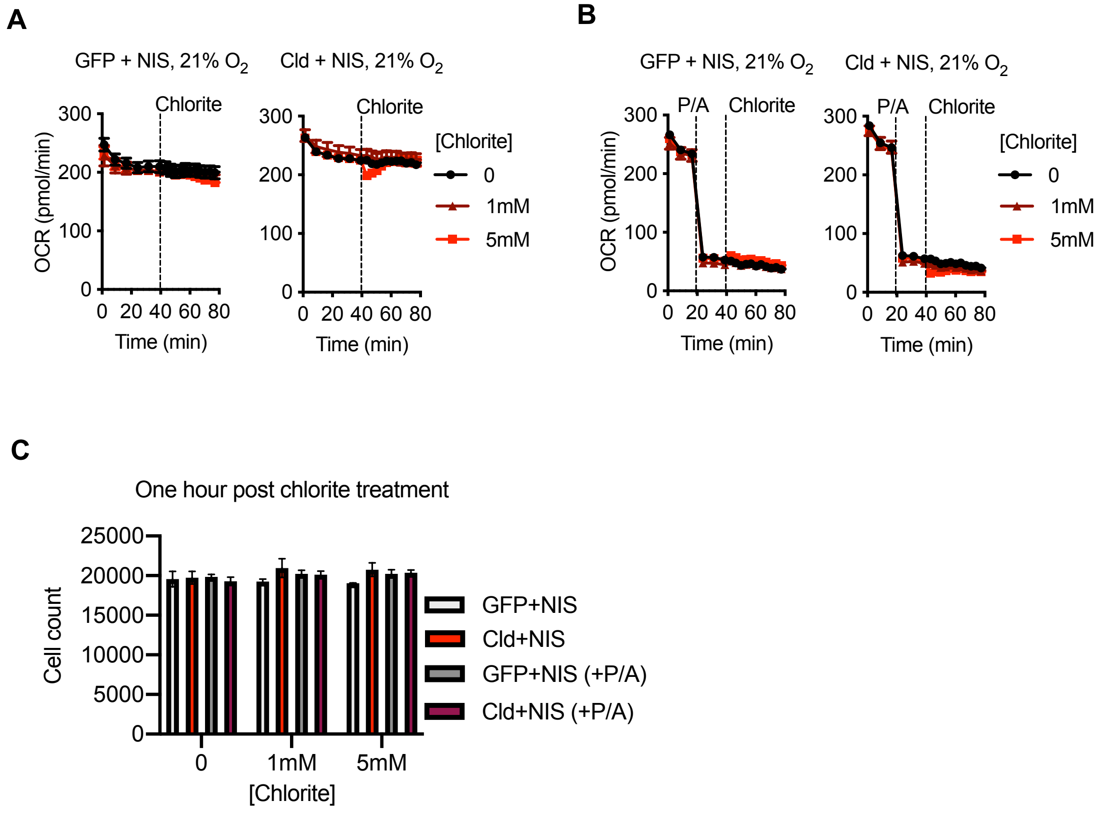

Figure S1

A. Seahorse intact cell oxygen consumption rate measurements at atmospheric 21% oxygen with addition sodium chlorite (0, 1 mM, or 5 mM) in HeLa cells expressing GFP + NIS or FLAG-*Nd*Cld + NIS. **B.** Seahorse intact cell oxygen consumption rate measurements at atmospheric 21% oxygen with sequential additions of piericidin+antimycin (1 μM each) and sodium chlorite (0, 1 mM, or 5 mM) in HeLa cells expressing GFP + NIS or FLAG-*Nd*Cld + NIS. **C.** Cell counts by Hoechst 33432 staining immediately after the Seahorse experiments, performed approximately 1 hour after chlorite addition. OCR: oxygen consumption rate; mean +/- s.e.m. of n = 4-6 biological replicates are shown in Fig S1a-b; means +/- s.d. of n = 4 samples are shown in Fig S1c.

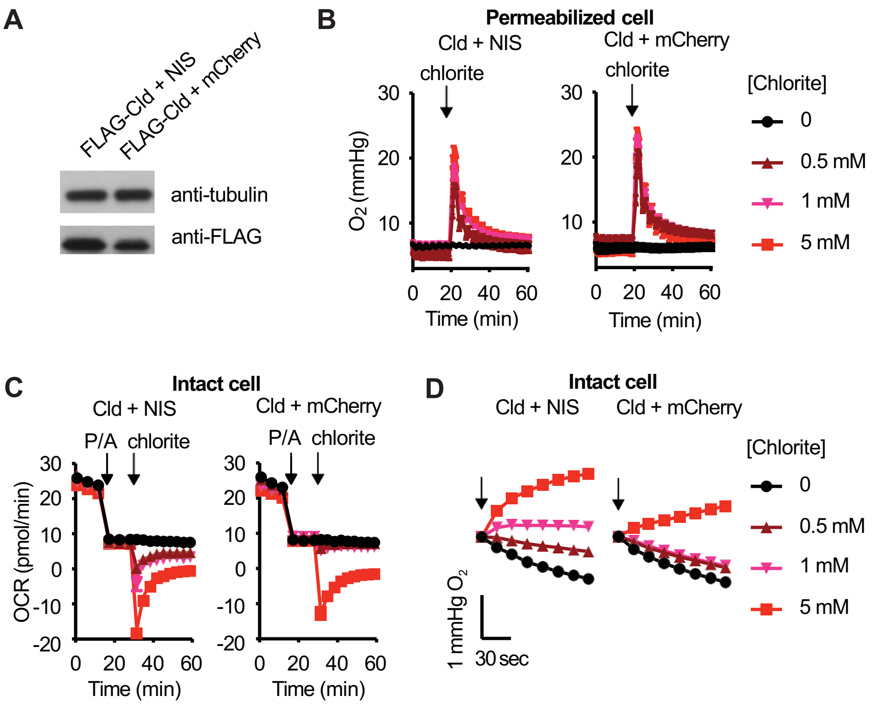

Figure S2

A. Immunoblot analysis of FLAG-*Nd*Cld in HeLa cells co-expressing NIS or mCherry. **B.** Seahorse permeabilized cell oxygen levels at 1% ambient oxygen with addition of sodium chlorite (0, 0.5 mM, 1 mM, or 5 mM) in HeLa cells expressing FLAG-*Nd*Cld + NIS or FLAG-*Nd*Cld + mCherry. **C.** Seahorse intact cell oxygen consumption rate measurements at 1% ambient oxygen with sequential additions of piericidin+antimycin (1 μM each) and sodium chlorite (0, 0.5 mM, 1 mM, or 5 mM) in HeLa cells expressing FLAG-*Nd*Cld + NIS or FLAG-*Nd*Cld + mCherry. **D.** Traces of the oxygen levels within two minutes upon sodium chlorite addition (black arrow) in the Seahorse experiments shown in Fig. S2c. OCR: oxygen consumption rate; mean +/- s.e.m. of n = 4-6 biological replicates are shown in Fig S2b-d.

Supporting Information Text

Supporting Methods. Listed below are the human codon optimized DNA and corresponding protein sequences used in this study.

FLAG-*Nd*Cld:
GCTAGCATGGATTACAAGGATGACGATGACAAGGGTGGATCTGGTGGATCTGGTGGATCTGCCGACCGGG
AAAAGCTGCTGACCGAGAGCGGTGTTTACGGCACATTCGCTACATTTCAGATGGACCATGATTGGTGGGA
CCTGCCTGGCGAATCCAGAGTGATCAGCGTGGCTGAAGTGAAGGGCCTGGTCGAGCAGTGGAGCGGAAAG
ATCCTGGTGGAATCTTATCTGCTGAGAGGCCTGAGCGACCACGCCGATCTGATGTTCAGAGTGCACGCCA
GAACCCTGTCTGATACCCAGCAGTTCCTGAGCGCCTTTATGGGCACCAGGCTGGGCAGACACCTGACCAG
CGGAGGACTTCTGCACGGCGTGTCCAAGAAACCTACATACGTGGCCGGCTTCCCCGAGTCTATGAAAACA
GAGCTGCAGGTCAACGGCGAGAGCGGCAGCAGACCTTACGCCATCGTGATTCCTATCAAGAAGGACGCCG
AATGGTGGGCCCTGGACCAGGAGGCCAGAACAGCCCTGATGCAGGAGCACACCCAGGCAGCTCTGCCATA
CCTGAAGACCGTGAAAAGAAAGCTGTACCACAGCACCGGCCTGGACGACGTGGACTTCATCACCTACTTC
GAGACAGAGCGGCTGGAAGATTTTCACAACCTGGTGCGGGCCCTGCAACAAGTGAAGGAGTTCAGACACA
ATCGGCGCTTCGGCCACCCTACCCTGCTGGGCACCATGAGCCCCCTGGATGAGATCCTCGAGAAGTTCGC
CCAGTGAGAATTC

MDYKDDDDKGGSGGSGGSADREKLLTESGVYGTFATFQMDHDWWDLPGESRVISVAEVKGLVEQWSGKIL
VESYLLRGLSDHADLMFRVHARTLSDTQQFLSAFMGTRLGRHLTSGGLLHGVSKKPTYVAGFPESMKTEL
QVNGESGSRPYAIVIPIKKDAEWWALDQEARTALMQEHTQAALPYLKTVKRKLYHSTGLDDVDFITYFET
ERLEDFHNLVRALQQVKEFRHNRRFGHPTLLGTMSPLDEILEKFAQ*

FLAG-*Nd*Cld^{4MUT}:
GCTAGCATGGATTACAAGGATGACGATGACAAGGGTGGATCTGGTGGATCTGGTGGATCTGCCGACCGGG
AAAAGCTGCTGACCGAGAGCGGTGTTTACGGCACATTCGCTACATTTCAGATGGACCATGATTGGTGGGA
CCTGCCTGGCGAATCCAGAGTGATCAGCGTGGCTGAAGTGAAGGGCCTGGTCGAGCAGTGGAGCGGAAAG
ATCCTGGTGGAATCTTATCTGCTGAGAGGCCTGAGCGACCACGCCGATCTGATGTTCAGAGTGCACGCCA
GAACCCTGTCTGATACCCAGCAGTTCCTGGCCGCCTTTATGAACACCAGGCTGGGCAGACACCTGACCGA
CGGAGGACTTCTGCACGGCGTGTCCAAGAAACCTACATACGTGGCCGGCTTCCCCGAGTCTATGAAAACA
GAGCTGCAGGTCAACGGCGAGAGCGGCAGCAGACCTTACGCCATCGTGATTCCTATCAAGAAGGACGCCG
AATGGTGGATGCTGGACCAGGAGGCCAGAACAGCCCTGATGCAGGAGCACACCCAGGCAGCTCTGCCATA
CCTGAAGACCGTGAAAAGAAAGCTGTACCACAGCACCGGCCTGGACGACGTGGACTTCATCACCTACTTC
GAGACAGAGCGGCTGGAAGATTTTCACAACCTGGTGCGGGCCCTGCAACAAGTGAAGGAGTTCAGACACA
ATCGGCGCTTCGGCCACCCTACCCTGCTGGGCACCATGAGCCCCCTGGATGAGATCCTCGAGAAGTTCGC
CCAGTGAGAATTC

- AGC – S110A – GCC
- GGC – G114N – AAC
- AGC – S123D – GAC
- GCC – A173M – ATG

MDYKDDDDKGGSGGSGGSADREKLLTESGVYGTFATFQMDHDWWDLPGESRVISVAEVKGLVEQWSGKIL
VESYLLRGLSDHADLMFRVHARTLSDTQQFLAAFMNTRLGRHLTDGGLLHGVSKKPTYVAGFPESMKTEL
QVNGESGSRPYAIVIPIKKDAEWWMLDQEARTALMQEHTQAALPYLKTVKRKLYHSTGLDDVDFITYFET
ERLEDFHNLVRALQQVKEFRHNRRFGHPTLLGTMSPLDEILEKFAQ*

FLAG-*Da*Cld:
GCTAGCATGGATTACAAGGATGACGATGACAAGGGTGGATCTGGTGGATCTGGTGGATCTCAGCAGGCCA
TGCAGCCCATGCAGAGCATGAAAATCGAGAGAGGAACCATCCTGACCCAGCCTGGCGTGTTCGGCGTCTT

TACCATGTTCAAGCTGCGCCCCGATTGGAACAAAGTGCCTGTTGCTGAGAGAAAAGGCGCCGCTGAGGAA
GTGAAAAAGCTGATCGAGAAGCACAAGGACAACGTGCTGGTCGACCTTTATCTGACCAGAGGCCTGGAAA
CCAACAGCGACTTTTTCTTCAGAATCAACGCCTACGACCTGGCCAAGGCCCAAACATTCATGAGAGAGTT
CCGGAGCACCACCGTGGGCAAGAACGCCGATGTGTTTGAGACCCTGGTCGGCGTGACCAAGCCTCTGAAT
TACATCAGCAAGGATAAGTCCCCAGGCCTCAACGCCGGCCTGTCTAGCGCTACATACAGCGGCCCTGCCC
CTAGATACGTGATCGTGATTCCTGTGAAGAAAAATGCTGAATGGTGGAATATGAGCCCCGAAGAGCGGCT
GAAGGAGATGGAAGTGCACACAACCCCTACCCTGGCCTACCTGGTGAACGTGAAGAGAAAGCTGTACCAC
AGCACTGGCCTGGACGACACCGACTTCATCACCTACTTCGAGACAGATGACCTGACCGCCTTCAACAACC
TGATGCTGTCTCTGGCCCAGGTGAAGGAAAACAAGTTCCACGTGCGGTGGGGATCTCCAACAACACTGGG
AACAATCCATTCTCCTGAGGACGTGATCAAGGCCCTGGCAGATTGAGAATTC

MDYKDDDDKGGSGGSGGSQQAMQPMQSMKIERGTILTQPGVFGVFTMFKLRPDWNKVPVAERKGAAEEVK
KLIEKHKDNVLVDLYLTRGLETNSDFFFRINAYDLAKAQTFMREFRSTTVGKNADVFETLVGVTKPLNYI
SKDKSPGLNAGLSSATYSGPAPRYVIVIPVKKNAEWWNMSPEERLKEMEVHTTPTLAYLVNVKRKLYHST
GLDDTDFITYFETDDLTAFNNLMLSLAQVKENKFHVRWGSPTTLGTIHSPEDVIKALAD*

*Nw*Cld-FLAG:

GCTAGCATGACTTTTACCGTGTTCACCGGCGGCGATAGCGGCGCCTGGTCCATCCTGAGCGTGGCCCCAG
TGATCGGCGAAAGCCTGATGGCCGCTTCTCATCTGGCTATCGCCCCTAGCCTCAGCCTGGGCGACACCAG
CGCCACCACCCCTTGGCAACTGAGAGGCGTCGCCAGCCACGCCCGCTACGTGGAAAGAGCCGAGAAGATC
GCCCTTACATCTGTGCAGGCCGGCCTGGGAAGAAACGAGGCCACAAGAGCTGCTCTGATCCCCATCAGAA
AGTCCGCCGCCTGGTGGGAGATGACCCAGGACGAGAGGCGGGCAATTTTCGAAGATAAGAGCCACCACAT
CGCTGCCAGCCTGAAATACCTGCCTGCCATCGCCAGACAGCTGTATCACTGCAGAGATATCGGAGAACCC
TTTGACTTCCTGACATGGTTCGAGTACGCCCCTGAGCACGCCACAATGTTCGAGGACCTGGTGGGCGTGC
TGCGGGCCACCGAGGAATGGACCTACGTTGAGCGGGAAGTGGACATCCGGCTGGCCAGAGCCATCGGTGG
ATCTGGTGGATCTGGTGGATCTGATTACAAGGATGACGATGACAAGTAAGAATTC

MTFTVFTGGDSGAWSILSVAPVIGESLMAASHLAIAPSLSLGDTSATTPWQLRGVASHARYVERAEKIAL
TSVQAGLGRNEATRAALIPIRKSAAWWEMTQDERRAIFEDKSHHIAASLKYLPAIARQLYHCRDIGEPFD
FLTWFEYAPEHATMFEDLVGVLRATEEWTYVEREVDIRLARAIGGSGGSGGSDYKDDDDK*

Mito-*Nw*Cld-FLAG:

GCTAGCATGAGCGTGCTCACCCCACTCCTGCTGCGGGGGCTGACCGGCAGCGCTACTTTTACCGTGTTCA
CCGGCGGCGATAGCGGCGCCTGGTCCATCCTGAGCGTGGCCCCAGTGATCGGCGAAAGCCTGATGGCCGC
TTCTCATCTGGCTATCGCCCCTAGCCTCAGCCTGGGCGACACCAGCGCCACCACCCCTTGGCAACTGAGA
GGCGTCGCCAGCCACGCCCGCTACGTGGAAAGAGCCGAGAAGATCGCCCTTACATCTGTGCAGGCCGGCC
TGGGAAGAAACGAGGCCACAAGAGCTGCTCTGATCCCCATCAGAAAGTCCGCCGCCTGGTGGGAGATGAC
CCAGGACGAGAGGCGGGCAATTTTCGAAGATAAGAGCCACCACATCGCTGCCAGCCTGAAATACCTGCCT
GCCATCGCCAGACAGCTGTATCACTGCAGAGATATCGGAGAACCCTTTGACTTCCTGACATGGTTCGAGT
ACGCCCCTGAGCACGCCACAATGTTCGAGGACCTGGTGGGCGTGCTGCGGGCCACCGAGGAATGGACCTA
CGTTGAGCGGGAAGTGGACATCCGGCTGGCCAGAGCCATCGGTGGATCTGGTGGATCTGGTGGATCTGAT
TACAAGGATGACGATGACAAGTAAGAATTC

MSVLTPLLLRGLTGSATFTVFTGGDSGAWSILSVAPVIGESLMAASHLAIAPSLSLGDTSATTPWQLRGV
ASHARYVERAEKIALTSVQAGLGRNEATRAALIPIRKSAAWWEMTQDERRAIFEDKSHHIAASLKYLPAI
ARQLYHCRDIGEPFDFLTWFEYAPEHATMFEDLVGVLRATEEWTYVEREVDIRLARAIGGSGGSGGSDYK
DDDDK*

Human SLC5A5:

GCTAGCATGGAAGCCGTGGAAACAGGCGAGAGACCTACATTCGGCGCTTGGGATTACGGCGTCTTCGCCC
TGATGCTGCTGGTGTCCACCGGCATCGGCCTGTGGGTGGGCCTGGCCAGAGGCGGCCAGCGGTCTGCCGA

GGACTTCTTCACCGGCGGCAGGCGGCTGGCCGCTCTGCCTGTGGGCCTGAGCCTGAGCGCCAGCTTCATG
TCTGCCGTTCAGGTACTGGGCGTTCCTTCTGAGGCCTACCGGTACGGCCTGAAGTTCCTGTGGATGTGCC
TGGGCCAGCTGCTGAACAGCGTGCTGACCGCCCTGCTGTTCATGCCTGTGTTTTACAGACTGGGCCTGAC
AAGCACCTATGAGTACCTGGAAATGAGATTCTCCAGGGCCGTGCGGCTGTGCGGCACCCTGCAATACATC
GTGGCAACAATGCTGTACACCGGAATCGTCATTTACGCCCCTGCCCTGATCCTGAATCAGGTGACCGGAC
TGGATATCTGGGCCTCTCTGCTGAGCACAGGCATTATCTGCACCTTCTACACAGCCGTGGGCGGAATGAA
AGCCGTGGTGTGGACCGATGTGTTCCAGGTTGTGGTGATGCTGAGCGGGTTTTGGGTGGTCCTGGCCAGA
GGCGTGATGCTGGTCGGAGGGCCAAGACAGGTGCTGACCCTGGCTCAGAACCACAGCAGAATCAACCTGA
TGGATTTCAACCCCGACCCCAGAAGCAGATACACATTTTGGACCTTTGTGGTGGGAGGCACCCTGGTGTG
GCTGTCTATGTACGGAGTGAATCAAGCCCAGGTGCAGAGATATGTGGCCTGCAGAACCGAGAAGCAGGCC
AAGCTGGCCCTGCTCATCAACCAGGTGGGCCTTTTCCTGATCGTCAGCAGCGCCGCCTGCTGCGGCATCG
TGATGTTCGTGTTCTACACCGACTGCGACCCCCTGCTCCTGGGCAGAATCTCCGCTCCAGACCAGTACAT
GCCCCTGCTGGTGCTGGACATCTTCGAGGACCTGCCTGGCGTGCCTGGATTGTTTCTGGCTTGTGCCTAC
AGCGGCACACTGAGCACCGCCAGCACCAGCATCAACGCCATGGCCGCCGTGACAGTGGAAGACCTGATTA
AACCCCGCCTGAGATCTCTGGCTCCTAGAAAGCTGGTTATCATCTCTAAGGGCCTGAGCCTGATCTACGG
CTCGGCGTGTCTGACCGTGGCCGCCTTGAGCAGCCTGCTGGGAGGCGGCGTGCTGCAGGGCAGCTTCACC
GTGATGGGCGTGATCAGCGGCCCTCTGCTCGGAGCATTCATCCTGGGCATGTTCCTGCCTGCCTGCAACA
CCCCTGGCGTACTCGCCGGCCTGGGCGCTGGACTGGCCCTGAGCCTCTGGGTGGCCCTGGGCGCTACACT
GTACCCCCCCAGCGAGCAGACCATGCGGGTGCTGCCATCCAGCGCCGCACGGTGCGTGGCCTTGTCCGTG
AACGCCTCTGGCCTCCTGGATCCTGCTCTTCTGCCTGCCAATGATAGCTCCAGAGCCCCTAGCAGCGGCA
TGGACGCCAGCAGGCCTGCCCTGGCTGATTCTTTCTATGCCATCAGCTACCTGTACTACGGCGCTCTGGG
CACCCTGACCACCGTGCTTTGTGGCGCCCTGATCAGCTGCCTGACTGGGCCTACCAAGCGGTCTACACTG
GCCCCTGGACTGCTGTGGTGGGACCTGGCCCGGCAGACAGCCAGCGTGGCCCCCAAGGAGGAAGTGGCTA
TCCTGGACGACAACCTGGTGAAGGGCCCGGAAGAGCTGCCCACCGGCAACAAGAAACCTCCAGGCTTCCT
CCCTACTAACGAGGACAGACTGTTTTTCCTGGGACAAAAGGAACTGGAAGGCGCCGGCAGCTGGACACCT
TGTGTGGGCCACGACGGCGGAAGAGACCAGCAGGAGACGAACCTGTGAGGTACC

MEAVETGERPTFGAWDYGVFALMLLVSTGIGLWVGLARGGQRSAEDFFTGGRRLAALPVGLSLSASFMSA
VQVLGVPSEAYRYGLKFLWMCLGQLLNSVLTALLFMPVFYRLGLTSTYEYLEMRFSRAVRLCGTLQYIVA
TMLYTGIVIYAPALILNQVTGLDIWASLLSTGIICTFYTAVGGMKAVVWTDVFQVVVMLSGFWVVLARGV
MLVGGPRQVLTLAQNHSRINLMDFNPDPRSRYTFWTFVVGGTLVWLSMYGVNQAQVQRYVACRTEKQAKL
ALLINQVGLFLIVSSAACCGIVMFVFYTDCDPLLLGRISAPDQYMPLLVLDIFEDLPGVPGLFLACAYSG
TLSTASTSINAMAAVTVEDLIKPRLRSLAPRKLVIISKGLSLIYGSACLTVAALSSLLGGGVLQGSFTVM
GVISGPLLGAFILGMFLPACNTPGVLAGLGAGLALSLWVALGATLYPPSEQTMRVLPSSAARCVALSVNA
SGLLDPALLPANDSSRAPSSGMDASRPALADSFYAISYLYYGALGTLTTVLCGALISCLTGPTKRSTLAP
GLLWWDLARQTASVAPKEEVAILDDNLVKGPEELPTGNKKPPGFLPTNEDRLFFLGQKELEGAGSWTPCV
GHDGGRDQQETNL*

mito-FLAG-*Nd*Cld:
GCTAGCATGCTCGCTACAAGGGTCTTTAGCCTCGTCGGAAAGAGAGCTATCAGCACCTCCGTCTGCGTGA
GAGCTCATGATTACAAGGATGACGATGACAAGGGTGGATCTGGTGGATCTGGTGGATCTGCCGACCGGGA
AAAGCTGCTGACCGAGAGCGGTGTTTACGGCACATTCGCTACATTTCAGATGGACCATGATTGGTGGGAC
CTGCCTGGCGAATCCAGAGTGATCAGCGTGGCTGAAGTGAAGGGCCTGGTCGAGCAGTGGAGCGGAAAGA
TCCTGGTGGAATCTTATCTGCTGAGAGGCCTGAGCGACCACGCCGATCTGATGTTCAGAGTGCACGCCAG
AACCCTGTCTGATACCCAGCAGTTCCTGAGCGCCTTTATGGGCACCAGGCTGGGCAGACACCTGACCAGC
GGAGGACTTCTGCACGGCGTGTCCAAGAAACCTACATACGTGGCCGGCTTCCCCGAGTCTATGAAAACAG
AGCTGCAGGTCAACGGCGAGAGCGGCAGCAGACCTTACGCCATCGTGATTCCTATCAAGAAGGACGCCGA
ATGGTGGGCCCTGGACCAGGAGGCCAGAACAGCCCTGATGCAGGAGCACACCCAGGCAGCTCTGCCATAC
CTGAAGACCGTGAAAAGAAAGCTGTACCACAGCACCGGCCTGGACGACGTGGACTTCATCACCTACTTCG
AGACAGAGCGGCTGGAAGATTTTCACAACCTGGTGCGGGCCCTGCAACAAGTGAAGGAGTTCAGACACAA
TCGGCGCTTCGGCCACCCTACCCTGCTGGGCACCATGAGCCCCCTGGATGAGATCCTCGAGAAGTTCGCC
CAGTGAGAATTC

MLATRVFSLVGKRAISTSVCVRAHDYKDDDDKGGSGGSGGSADREKLLTESGVYGTFATFQMDHDWWDLP
GESRVISVAEVKGLVEQWSGKILVESYLLRGLSDHADLMFRVHARTLSDTQQFLSAFMGTRLGRHLTSGG
LLHGVSKKPTYVAGFPESMKTELQVNGESGSRPYAIVIPIKKDAEWWALDQEARTALMQEHTQAALPYLK
TVKRKLYHSTGLDDVDFITYFETERLEDFHNLVRALQQVKEFRHNRRFGHPTLLGTMSPLDEILEKFAQ*
